# FOXK2 promotes the proliferation of papillary thyroid cancer cell by down-regulating autophagy

**DOI:** 10.7150/jca.60730

**Published:** 2022-01-01

**Authors:** Songze Li, Pengliang Wang, Hao Ju, Tiantong Zhu, Jingwen Shi, Ying Huang

**Affiliations:** 1 Department of Ultrasound, Shengjing Hospital of China Medical University, Shenyang, Liaoning 110004, China.; 2 Department of Anesthesiology, Cancer Hospital of China Medical University, Liaoning Cancer Hospital &Institute, Shenyang, Liaoning 110122, China.; 3 Department of Gastroenterology, Tianjin Medical University Cancer Hospital, City Key Laboratory of Tianjin Cancer Center and National Clinical Research Center for Cancer, Tianjin, China.

**Keywords:** FOXK2, proliferation, autophagy, papillary thyroid cancer

## Abstract

Papillary thyroid cancer (PTC) is the most common endocrine system tumor. FOXK2 is involved in the development of different types of cancers, however, its function has not been investigated in papillary thyroid cancer. In the present study, we demonstrated that FOXK2 expression was up-regulated in papillary thyroid carcinoma tissues compared with matched normal tissues. Importantly, we found that FOXK2 expression was significantly associated with the tumor size, T stage, and TNM stage. Furthermore, stable knockdown of FOXK2 markedly inhibited PTC cell proliferation, significantly increased the ratio of LC3-II/LC3-I, and reduced p62 expression, whereas overexpression of FOXK2 showed opposite effects. In FOXK2 knockdown cell lines, mCherry-GFP-LC3 immunofluorescence demonstrated increased punctate aggregates of mCherry-GFP-LC3, and transmission electron microscopy revealed increased numbers of autophagosomes. Autophagy-related protein ULK1, VPS34, and FOXO3 were markedly up-regulated by FOXK2 knockdown and down-regulated by FOXK2 overexpression. Finally, autophagy inhibitor 3-MA attenuated autophagy activation and rescued the inhibition of cell proliferation caused by FOXK2 knockdown, suggesting that FOXK2 silencing inhibits cell proliferation through up-regulating autophagy. These findings revealed an important role of FOXK2 in PTC progression and suggested that FOXK2 might be a potential new target for the diagnosis and treatment of PTC.

## Introduction

Thyroid cancer is one of the most common endocrine system tumors 1. From 1975 through 2016, the incidence of thyroid cancer showed a rising trend year after year 2, 3. About 90% of thyroid cancer is well-differentiated papillary thyroid cancer (PTC) 4. Although papillary thyroid carcinoma is usually indolent and has a good prognosis, the disease persistence and local recurrence are still the reasons for its increased mortality 5. Therefore, it is quite necessary to explore the molecular mechanism that promotes the progression of papillary thyroid carcinoma and to provide new treatment strategies.

FOXK2, as one of the important transcription factors of the Forkhead Box (FOX) family 6, has been shown to control a grouping number of biological functions, such as cell proliferation 7-9, apoptosis 10, DNA repair 11, as well as regulating metabolism 12, 13. More and more evidence show that FOXK2 is tightly related to the malignant process of tumors. For example, FOXK2 was up-regulated in colorectal cancer and promoted the malignant phenotype of colorectal cancer through regulating Wnt/beta-catenin signaling 14 or up-regulating the expression of ZEB1 and EGFR 15. In addition, FOXK2 regulated by miR-1271-5p increased cell proliferation and suggested a poor prognosis of liver cancer 16. However, the expression and biological function of FOXK2 have not been investigated in papillary thyroid carcinoma.

Autophagy is an evolutionarily conserved catabolic process that functions in nutrient recycling, energy generation, as well as the clearance of damaged proteins and organelles in a lysosome-dependent manner 17, 18. The "cargo" was firstly wrapped by a double-layer membrane structure to form autophagosomes, and then autophagosomes and lysosomes are fused to form autophagolysosomes, and the contents are degraded and recycled. Dysfunction of autophagy plays an important role in diseases development, especially in tumor formation 19-21. In the process of tumorigenesis, autophagy can inhibit the occurrence of tumors and inhibit the proliferation of tumor cells 22-26. However, autophagy can help tumor cells resist apoptosis when cells are faced with stress such as nutritional deficiency or chemotherapy 27-31. The regulation process of autophagy is coordinated by ATG protein 32, 33. Among them, it is well known that the ULK1 and Vps34 complexes play important regulatory roles in the initiation and development of autophagy 34.

In this study, we clarified the expression and clinical significance of FOXK2 in papillary thyroid carcinoma. In addition, we described the effects and underlying mechanisms of FOXK2 on autophagy in PTC cell lines and suggested that FOXK2 might be a potential therapeutic target for the treatment of PTC.

## Materials and Methods

### Patients and tissue specimens

The human papillary thyroid cancer specimens used in this study were collected from patients who were treated with surgery and pathologically diagnosed with papillary thyroid cancer in the Shengjing Hospital of China Medical University from 2011 to 2016. After the operation, we store the removed thyroid tissue in liquid nitrogen. This study was conducted with the approval of the Ethics Committee at the Institutional Review Board of Shengjing Hospital of China Medical University. Informed consent was obtained from all patients.

For Immunohistochemistry, five-micrometer-thick consecutive sections were exposed to anti-FOXK2 (1:100) antibody overnight at 4°C. After washes with PBS, sections were incubated in horseradish peroxidase-conjugated goat anti-rabbit immunoglobulin G (1:200) for 2 h, followed by development with 0.003% H_2_O_2_ and 0.03% 3, 3'-diaminobenzidine in 0.05 mol/L Tris-HCl. Five areas selected at random were scored. All sections were scored in a semiquantitative manner, which reflects both the intensity and percentage of cells staining at each intensity. Intensity was classified as 0 (no staining), +1 (weak staining), +2 (distinct staining) or +3 (very strong staining). A value designated as the 'HSCORE' was obtained for each slide by using the following algorithm: HSCORE=∑(I×PC), where I and PC represent the staining intensity and the percentage of cells that stain at each intensity, respectively. And the corresponding HSCOREs were calculated separately.

### Cell culture and viral infection

Nthy-ori 3-1 (a generous gift of Hao Zhang), TPC-1 (a generous gift of Bryan R. Haugen), K1 (The Health Protection Agency Culture Collections, UK), BCPAP (DSMZ, Braunschweig, Germany) and BHT101 (Procell, Wuhan, China) were cultured in RPMI 1640 medium supplemented with 10% fetal bovine serum (FBS) in the 37 °C incubator (5% CO_2_ atmosphere).

Cells were infected with commercialized FOXK2-silencing lentivirus (Shanghai GeneChem Company) and screened with puromycin for constructing a stable FOXK2-silencing cell line. The knockdown was performed with shFOXK2. The shRNA control sequence was 5'-TTCTCCGAACGTGTCACGTtt-3'; the FOXK2 sequence #1 was 5'- CCTCAATTTAATGGCTGACAA-3'; the FOXK2 sequence #2 was 5'-CGAGTTCGAGTATCTGATGAA -3'. siRNA specific for human ULK1, VPS34 and FOXO3 were purchased from RIBOBIO Co. (Guangzhou, China).

### Western blot

Protein was extracted from cells and tissues with RIPA lysis buffer (50 mM Tris-HCl(pH 7.4), 150 mM NaCl, 1% NP-40, 0.1% SDS). Protein concentrations were measured by BCA method (Beyotime, China). Antibodies against FOXK2 (#12008, 1:1,000 dilution), LC3A/B (#4108, 1:1,000 dilution), ULK1 (#8054, 1:1,000 dilution), FOXO3a (#2497, 1:1,000 dilution) were from Cell Signaling Technology. Antibodies against p62/SQSTM1 (P0067, 1:2,000 dilution) and VPS34 (V9764, 1:1,000 dilution) were from Sigma. Tubulin antibody (SG4110-16, 1:1000 dilution) was from Shanghai Genomics Technology.

### Cell proliferation assay and Colony formation assay

The proliferation rate of BHT101 (shRNA-NC, shRNA-FOXK2) and BCPAP (Lv-vector, Lv-FOXK2) cells were assessed using the Cell Counting Kit-8 (CCK-8) assay (Beyotime Biotechnology, China). Cells (2 × 10^3^ cells per well) were seeded into 96-well culture plates and cultured for 24, 48, 72 and 96 h. Then cells were incubated with 10 μl of CCK-8 solution for 3 h at 37 °C. The number of viable cells was calculated based on absorbance at 450 nm.

For colony formation assays, 7×10^2^ cells were plated in 6-well plates. The plates were incubated at 37°C in a 5% CO_2_ incubator. After two weeks, the plates were then stained using Giemsa.

### Fluorescence microscopy and electron Microscopy

For immunofluorescence analysis, after the knockdown FOXK2 cells infected with mCherry-GFP-LC3 lentivirus were grown on coverslips, cells were fixed in 4% paraformaldehyde for 10 mins and then permeabilized with 0.15% Triton X-100 for 10mins. Fixed cells were incubated with DAPI for 5 mins, and then coverslips were washed with phosphate-buffered saline (PBS) for three times. Cells were visualized under a microscope (Nikon, Ti-E, DS-Ri2).

For electron microscopy (EM) analyses, after the cells were trypsinized, washed once with PBS, and then fixed with 2.5% glutaraldehyde overnight. After fixation, cells were washed three times in PBS and then postfixed in aqueous 1% OsO_4_ and 1% K_3_Fe(CN)_6_ for 1 h. After three times PBS washes, the cell block was dehydrated and infiltrated with a gradient of 30%-100% ethanol, and then embedded in Polybed 812 epoxy resin. Ultrathin sections were collected on copper grids and stained with 2% uranyl acetate in 50% methanol for 10 min, followed by 1% lead citrate for 10 min. EM is executed by the Research Department of the Electron Microscope Center of China Medical University.

### Statistical analysis

We carried out statistical comparisons by using unpaired Student's t test, Pearson χ^2^ or the Mann-Whitney U test when a normal distribution could not be assumed. Three and more groups were compared by one-way ANOVA. Data are presented as mean ± s.e.m. We tested data for normality and variance, and considered a P value of less than 0.05 as significant. Statistical analyses were performed using SPSS software.

## Results

### FOXK2 was up-regulated in papillary thyroid cancer

**Figure 1:**
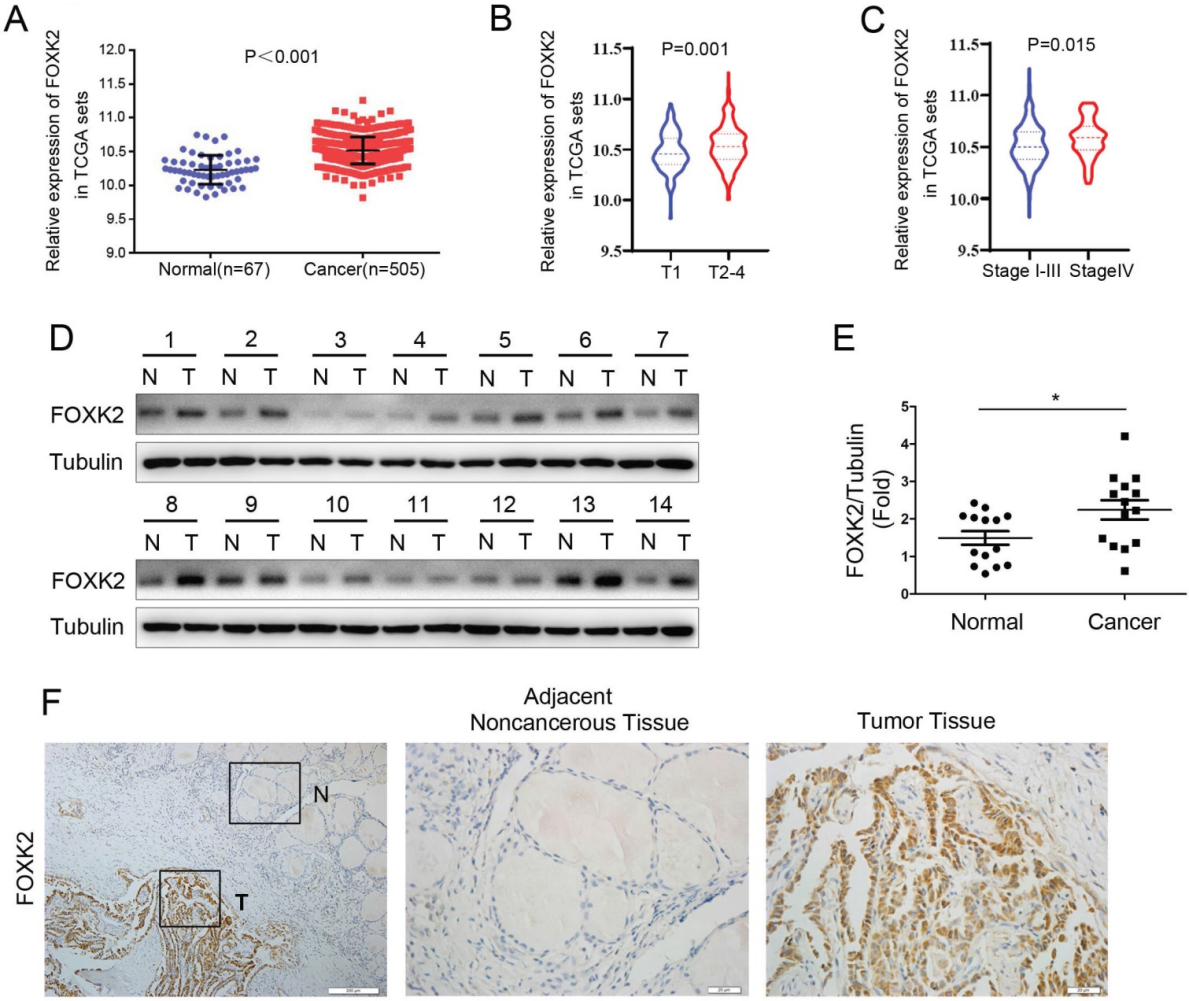
** FOXK2 was up-regulated in papillary thyroid cancer. (A)** The mRNA expression of FOXK2 in human papillary thyroid cancer tissues and normal thyroid tissues was analyzed by the TCGA cohort. **(B)** The mRNA expression of FOXK2 in human papillary thyroid cancer tissues according to the T stage was analyzed by the TCGA cohort. **(C)** The mRNA expression of FOXK2 in human papillary thyroid cancer tissues according to the TNM stage was analyzed by the TCGA cohort. **(D)** The protein expression of FOXK2 was analyzed by western blot analysis from four randomly selected PTC patients. Tubulin was used as a loading control. **(E)** The protein expression of FOXK2 was visualized via scatter diagram. * P < 0.05. **(F)** Representative immunohistochemical staining images of FOXK2 in the PTC and Adjacent noncancerous tissue. The image of the box area was magnified and displayed by the adjacent non-cancerous tissue (N) and tumor tissue (T).

To explore whether FOXK2 is involved in the malignant process of thyroid cancer, we first analyzed the expression of FOXK2 in thyroid cancer through the TCGA database. FOXK2 was significantly up-regulated in thyroid cancer tissues compared to normal tissues (Fig. 1A). Furthermore, We found that compared with the T1 stage group, the expression of FOXK2 in the T2-4 stage groups increased (P=0.001, unpaired Student's t test) (Fig. 1B), and compared with the T1-3 TNM stage groups, the expression of FOXK2 in the T4 TNM stage group increased (P=0.015, unpaired Student's t test) (Fig. 1C). Then Western blot analysis validated that FOXK2 expression was up-regulated in PTC tissues (n=14) compared to matched normal tissues (Fig. 1D and E). We also performed immunohistochemical staining of 98 pairs of PTC tissues and matched adjacent normal tissues to detect FOXK2 expression in PTC tissues. Among the 98 adjacent normal tissue samples, 89 (90.8%) were negative for FOXK2 expression. In contrast, FOXK2 expression was significantly higher in PTC tissues, and 69 of the 98 tumor samples (70.4%) were positive for FOXK2 staining (Fig. 1F). More importantly, through TCGA database analysis, we found that the expression of FOXK2 is closely related to the clinicopathological characteristics of thyroid cancer. The high expression of FOXK2 is associated with Tumor size (*P*=0.002), T stage (*P*=0.001), and TNM stage (*P*=0.016) (Table 1). Similarly, in our validated cohort, high FOXK2 expression was correlated with the tumor size (*P*=0.001) and tumor TNM stage (*P*= 0.013), as shown in Table 2. In summary, FOXK2 is highly expressed in papillary thyroid cancer and is closely related to its malignant progression.

### Knockdown of FOXK2 suppressed the proliferation of PTC cells

**Figure 2:**
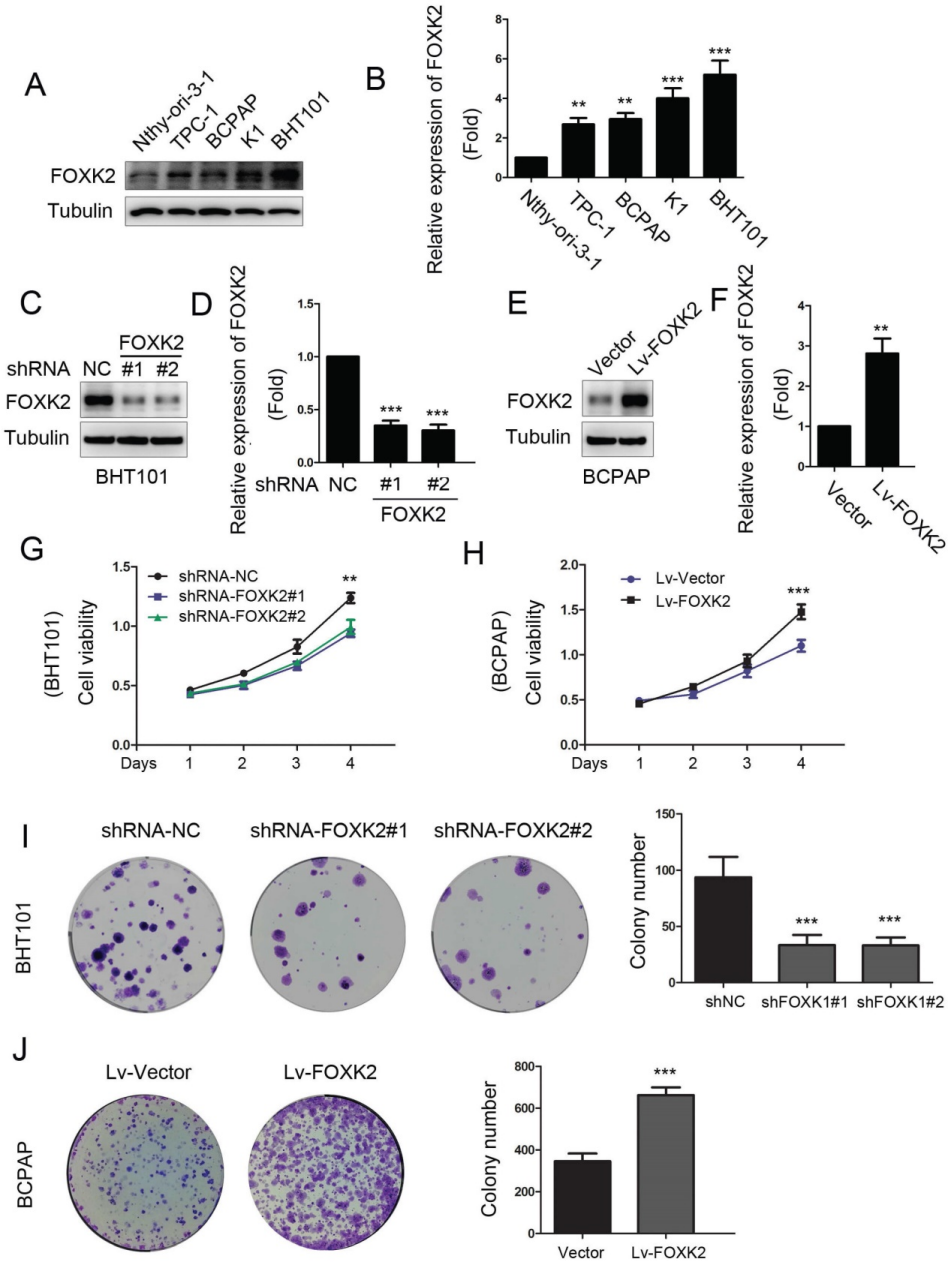
** Knockdown of FOXK2 suppressed the proliferation of PTC cells. (A)** Western blot detection of FOXK2 expression in human normal thyroid follicular epithelial cells and papillary thyroid cancer cell lines. Tubulin was used as a loading control. **(B)** The protein expression of FOXK2 in human normal thyroid follicular epithelial cells and papillary thyroid cancer cell lines was visualized via quantitative analysis. ** P < 0.01; ***P < 0.001. **(C)** Western blot detection of FOXK2 in BHT-101 cells transfected with indicated plasmids. **(D)** The protein expression of FOXK2 in BHT-101 cells was visualized via quantitative analysis. ***P < 0.001. **(E)** Western blot detection of FOXK2 in BCPAP cells transfected with indicated plasmids. **(F)** The protein expression of FOXK2 in BCPAP cells was visualized via quantitative analysis. ** P < 0.01. **(G, H)** The ability of cell proliferation was monitored by daily quantification in BHT-101 and BCPAP cell lines transfected with indicated plasmids for up to 4 days. ** P < 0.01; ***P < 0.001. **(I, J)** The ability of cell colony formation was detected in BHT-101 and BCPAP cell lines transfected with indicated plasmids**.** ***P < 0.001.

To characterize the biological functions of FOXK2 in PTC, we first detected the expression of FOXK2 in PTC cell lines. We found that the expression of FOXK2 was higher in most the PTC cell lines than that in human thyroid follicular epithelial cells Nthy-ori-3-1 (Fig. 2A and B). Based on the relative high and low expression of FOXK2 in papillary thyroid cancer cell lines, we constructed RNAi-mediated knockdown PTC cells in BHT-101 and FOXK2-overexpressed cell lines in BCPAP. Transfection efficiency was examined by Western blot (Fig. 2C-F). Using CCK8 and colony formation experiments, we found that knockdown of FOXK2 can inhibit the proliferation and colony formation in BHT-101 cells (Fig. 2G and I). On the contrary, overexpression of FOXK2 can promote the proliferation and colony formation in BCPAP cells (Fig. 2H and J).

### Knockdown of FOXK2 promoted autophagy of PTC cells

**Figure 3:**
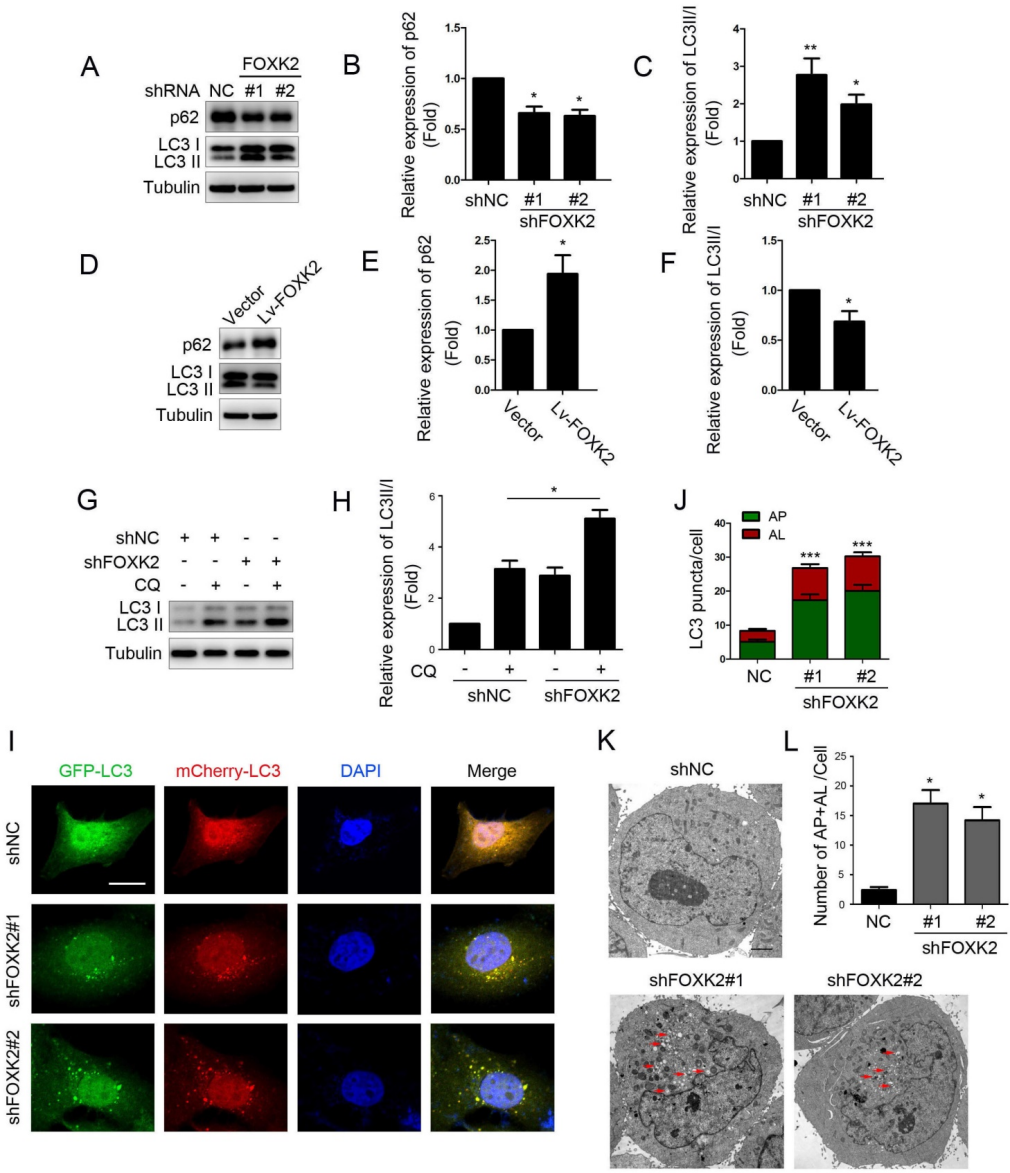
** Knockdown of FOXK2 promoted autophagy of PTC cells. (A)** Western blot detection of p62 and LC3 in BHT-101 cells transfected with indicated plasmids. **(B, C)** The protein expression of p62 (B) and LC3II/I (C) in BHT-101 cells was visualized via quantitative analysis. * P < 0.05; **P < 0.01. **(D)** Western blot detection of p62 and LC3 in BCPAP cells transfected with indicated plasmids. **(E, F)** The protein expression of p62 (E) and LC3II/I (F) in BCPAP cells was visualized via quantitative analysis. * P < 0.05. **(G)** Western blot detection of LC3 in BHT-101 cells transfected with the indicated shRNA, pre-treated with 60nM CQ or vehicle (DMSO). **(H)** The protein expression of LC3II/I in BHT-101 cells was visualized via quantitative analysis. *P < 0.05. **(I)** Autophagic flux is shown by representative confocal microscopic images of BHT-101 cells stably-expressing GFP-mCherry-LC3 transfected with the indicated shRNA. Scale bar, 10 μm. **(J)** Quantitation of autophagosomal (yellow) and autolysosomal (red) LC3 puncta. Results are presented as mean ± s.e.m. from three independent experiments, ***P < 0.001. **(K)** Representative electron microscopic image of autophagic vesicles in BHT-101 cells transfected with the indicated shRNA. Scale bars, 500 nm. **(L)** Electron microscopic quantification of autophagic vesicles in BHT-101 cells transfected with the indicated shRNA. Data are presented as mean±s.e.m. from 3 independent experiments; *P < 0.05.

**Figure 4:**
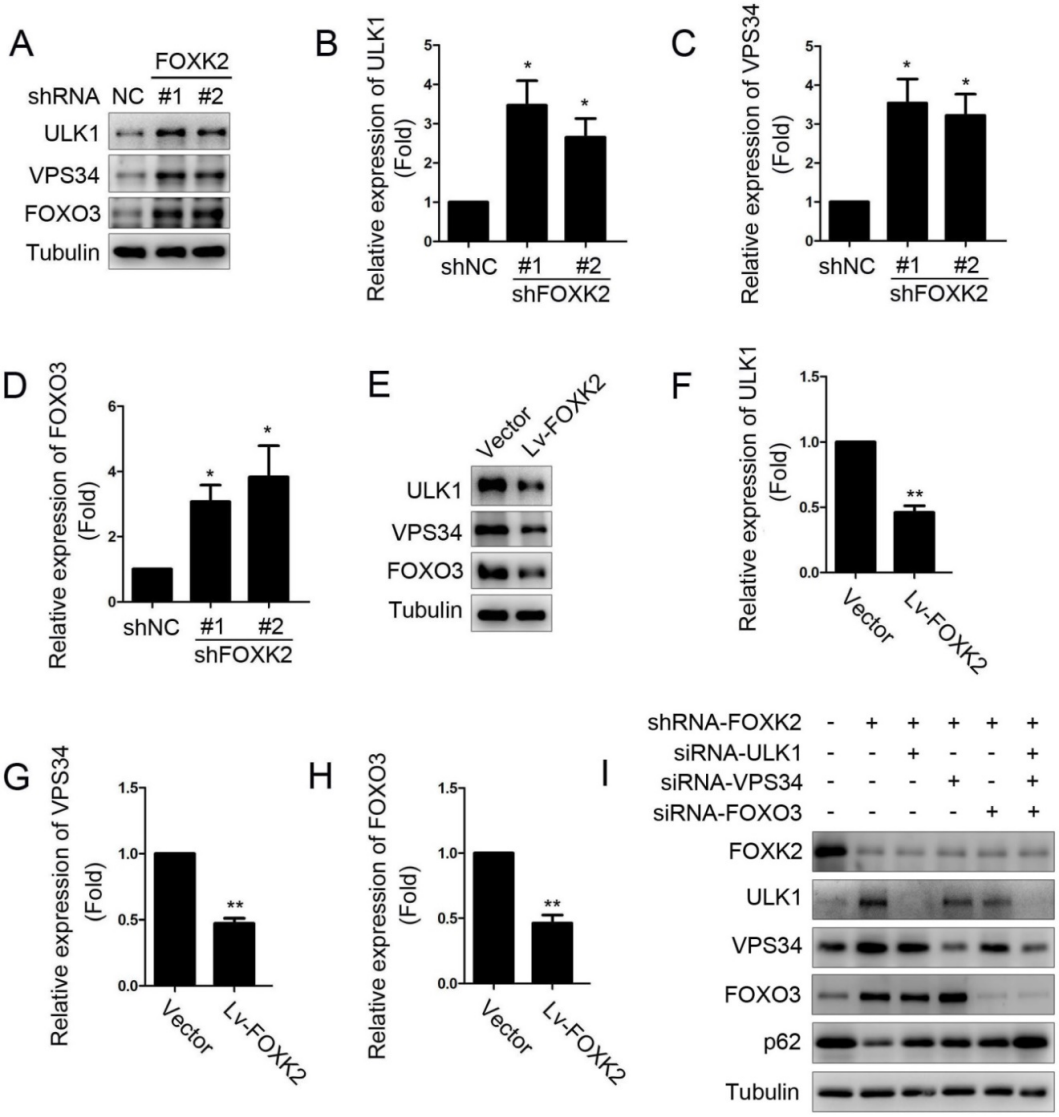
** Knockdown of FOXK2 promoted the expression of autophagy-related protein ULK1, VPS34 and FOXO3. (A)** Western blot detection of ULK1, VPS34 and FOXO3 in BHT-101 cells transfected with indicated plasmids. **(B-D)** The protein expression of ULK1 (B), VPS34 (C) and FOXO3 (D) in BHT-101 cells was visualized via quantitative analysis. * P < 0.05. **(E)** Western blot detection of ULK1, VPS34 and FOXO3 in BCPAP cells transfected with indicated plasmids. **(F-H)** The protein expression of ULK1 (F), VPS34 (G) and FOXO3 (H) in BCPAP cells was visualized via quantitative analysis. ** P < 0.01. **(I)** Western blot detection of FOXK2, ULK1, VPS34, FOXO3 and p62 in BCPAP cells transfected with indicated plasmids and siRNA.

Next, we want to explore the possible molecular mechanism of FOXK2 affecting cell proliferation. Given that autophagy is an important cell biological behavior affecting tumorigenesis and tumor proliferation, we tested whether FOXK2 is involved in the regulation of autophagy levels in PTC cells. Using Western blotting, we found that silencing of FOXK2 promoted autophagy in BHT-101 cells (Fig. 3A-C), as demonstrated by the decrease of autophagy substrate p62 and increase of the conversion of the nonlipidated form (LC3-I) to the phosphatidyl ethanolamine-conjugated form (LC3-II) of LC3. Conversely, overexpression of FOXK2 suppressed autophagy levels in BCPAP cells (Fig. 3D-F). CQ, an inhibitor of autophagy, markedly blocked FOXK2 knockdown-induced autophagy flux and increased the accumulation of LC3-II, indicating an accelerated conversion of LC3-I to LC3-II (Fig. 3G and H). Next, we examined mCherry-GFP-LC3 puncta to assess autophagosome formation in the nonspecific RNAi-treated or FOXK2 knockdown BHT-101 cells. We found that knockdown of FOXK2 promoted the number of autophagosomes (Fig. 3I and J). In addition, quantitative electron microscopic analysis showed that knockdown of FOXK2 increased autophagic structures compared with the shRNA negative control cells (Fig 3K and L). Furthermore, we found that silencing of FOXK2 can promote the expression of critical autophagy proteins ULK1, VPS34, and FOXO3 (Fig 4A-D). Conversely, overexpression of FOXK2 suppressed protein levels of ULK1, VPS34, and FOXO3 in BCPAP cells (Fig 4E-H). Furthermore, silencing of ULK1, VPS34 and FOXO3 attenuated autophagy activation caused by FOXK2 knockdown at the protein level (Fig. 4I). Taken together, the silencing of FOXK2 might promote autophagy by up-regulating the expression of ULK1, VPS34, and FOXO3 in PTC cells.

### FOXK2 knockdown inhibited the proliferation of PTC cells through its regulating autophagy

**Figure 5:**
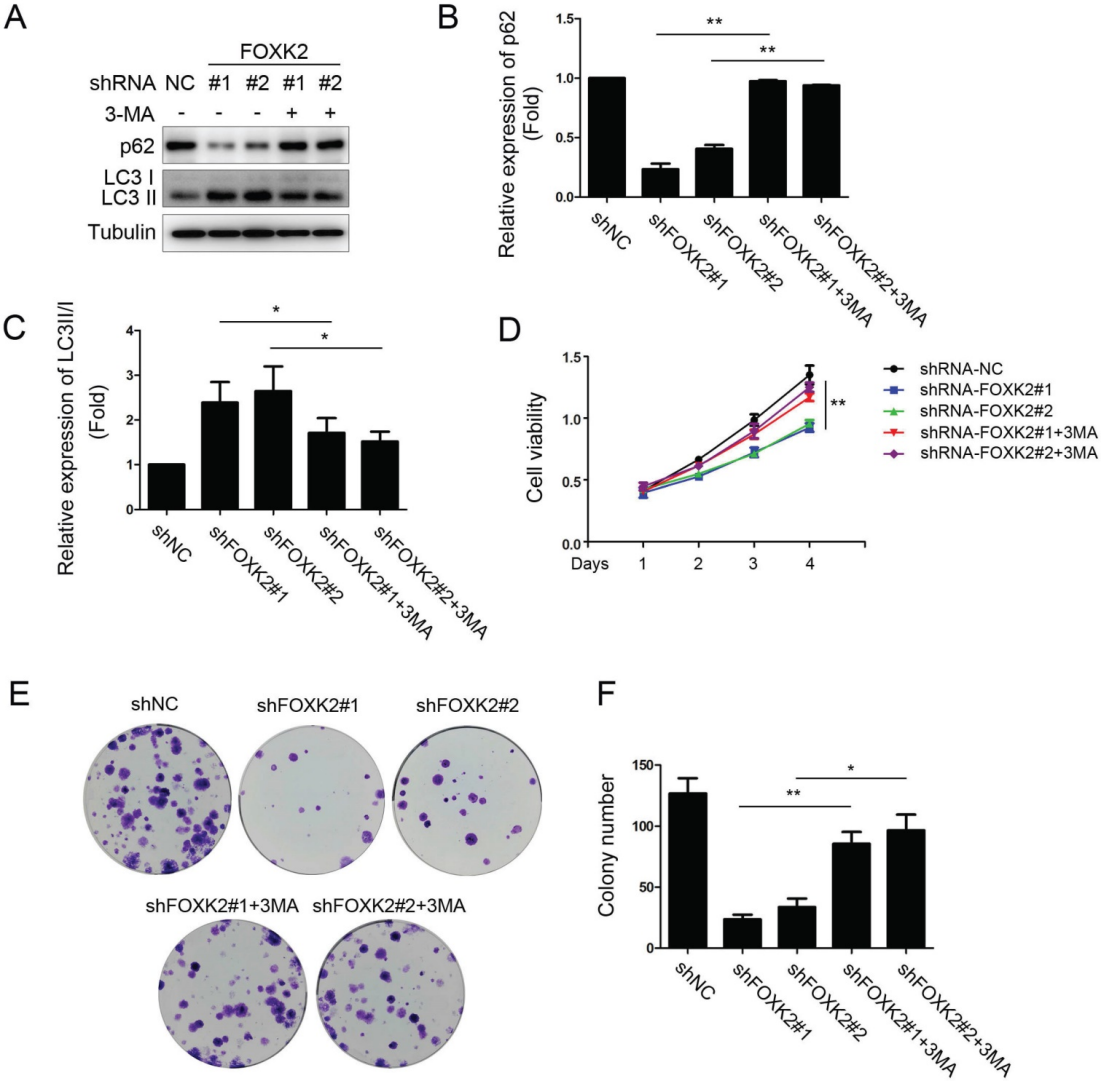
** FOXK2 knockdown inhibited the proliferation of PTC cells through its regulating autophagy**. **(A)** Western blot detection of p62 and LC3 in BHT-101 cells transfected with indicated plasmids with or without 3-MA. Tubulin was used as a loading control. **(B)** The protein expression of p62 in BHT-101 cells treated with 3-MA was visualized via quantitative analysis. ** P < 0.01. **(C)** The protein expression of LC3II/I in BHT-101 cells treated with 3-MA was visualized via quantitative analysis. *P < 0.05. **(D)** The ability of cell proliferation was monitored by daily quantification in BHT-101 cells transfected with indicated plasmids for up to 4 days with or without 3-MA. ** P < 0.01. **(E-F)** The ability of cell colony formation was detected in BHT-101 cell lines transfected with indicated plasmids with or without 3-MA**.** * P < 0.05; **P < 0.01.

To further study the relationship between FOXK2 knockdown-induced autophagy activation and cell proliferation associated with autophagy in PTC cell lines, we treated PTC cell lines silencing FOXK2 with autophagy inhibitor 3-MA. We found that 3-MA attenuated autophagy activation as demonstrated by the increase of autophagy substrate p62 and decrease of the conversion of LC3-I to LC3-II in BHT-101 PTC cells (Fig. 5A-C) and reversed the inhibition of cell proliferation and colony formation caused by silencing FOXK2 (Fig. 5D-F). In summary, these findings suggested that the silencing of FOXK2 inhibited cell proliferation and colony formation through up-regulating autophagy.

## Discussion

In recent years, there has been more and more evidence about the role of FOXK2 in tumors, but FOXK2 may play the opposite role in different tumors. Many studies have reported that FOXK2 can act as a tumor suppressor gene to inhibit the progression of breast cancer 35, 36, non-small cell lung cancer 7, and glioma 37. On the contrary, the expression of FOXK2 is closely related to the occurrence and development of tumors. For example, researchers have found that FOXK2 can promote the malignant phenotype of colorectal cancer through regulating Wnt/beta-catenin signaling 14 or up-regulating the expression of ZEB1 and EGFR 15. In addition, FOXK2 regulated by miR-1271-5p increased cell proliferation and suggested a poor prognosis of liver cancer 16. These contradictory findings indicate that whether FOXK2 plays an oncogene or a tumor suppressor in tumor progression is tumor-specific. However, the expression and biological function of FOXK2 have not been investigated in papillary thyroid carcinoma.

Thyroid cancer is one of the most common endocrine system malignancies. It is estimated that by 2030, its incidence will rise to become the fourth most common cancer 38. Therefore, exploring the biomarkers of thyroid cancer and the molecular mechanism and action rules of its occurrence and development will become an important scientific issue in the field of thyroid cancer research. In this study, we for the first time reported that FOXK2 plays an important role in papillary thyroid cancer as an oncogene and promotes the malignant progression of tumors. We showed that FOXK2 was up-regulated in PTC samples and cell lines. We found that the expression of FOXK2 was significantly up-regulated in the PTC tissues compared with the expression in the adjacent normal thyroid tissues in both the validation and TCGA cohorts. More importantly, increased levels of FOXK2 were found to significantly correlate with tumor size and tumor TNM stage. These findings suggested that FOXK2 is closely related to the malignant process of clinical PTC.

Proliferation is an important feature of tumor cells and a necessary condition for tumor malignant progression 39. To characterize the biological functions of FOXK2 in PTC, we first detected the expression of FOXK2 in PTC cell lines. We found that the expression of FOXK2 was higher in most the PTC cell lines than that in human thyroid follicular epithelial cells Nthy-ori-3-1. Furthermore, we found that knockdown of FOXK2 can inhibit the proliferation and colony formation in BHT-101 cells, and overexpression of FOXK2 can promote the proliferation and colony formation in BCPAP cells.

Dysfunction of autophagy plays an important role in diseases development, especially in tumor formation 40, 41. In the process of tumorigenesis, autophagy can inhibit the occurrence of tumors and inhibit the proliferation of tumor cells 42, 43. A previous study found that FOXK2 can inhibit the expression of critical autophagy genes by recruiting the Sin3A-HDAC complex and limiting the acetylation of histone H4 44. In addition, FOXK2 phosphorylation mediated by cell cycle checkpoint kinase 2 inhibits autophagy in a transcriptionally regulated manner 45. Consistent with previous studies, our results show that silencing of FOXK2 might promote autophagy by up-regulating the expression of critical autophagy proteins ULK1, VPS34, and FOXO3. Furthermore, autophagy inhibitor 3-MA attenuated autophagy activation and reversed the inhibition of cell proliferation and colony formation caused by silencing FOXK2, which suggested that silencing of FOXK2 inhibited cell proliferation and colony formation through up-regulating autophagy.

In conclusion, we described the molecular mechanism of FOXK2 as an oncogene in papillary thyroid cancer, which promotes cell proliferation and colony formation by inhibiting autophagy.

## Figures and Tables

**Table 1:** The relationship between FOXK2 and clinicopathologic characteristics in the TCGA cohort.

Clinicopathologic characteristics	Low expression (%)	High expression (%)	χ^2^	P
Age (N=505)			1.149	0.284
<55	175(69.2%)	163(64.7%)		
≥55	78 (30.8%)	89(35.3%)		
Gender (N=505)			0.140	0.708
Female	183(72.3%)	186(73.8%)		
Male	70(27.7%)	66(26.2%)		
Histological type (N=505)			6.455	0.091
Classical	181(71.5%)	177(70.2%)		
Follicular	57(22.5%)	45(17.9%)		
Tall Cell	12(4.7%)	24(9.5%)		
other	3(1.2%)	6(2.4%)		
Tumor size (N=405)			10.000	0.002
<2cm	95(38.6%)	62(25.3%)		
≥2cm	151(61.4%)	183(74.7%)		
T stage (N=503)			10.458	0.001
T1	88(34.9%)	55(21.9%)		
>T1	164(65.1%)	196(78.1%)		
Extrathyroidal invasion (N=487)			2.493	0.114
NO	172(72.0%)	162(65.3%)		
YES	67(28.0%)	86(34.7%)		
Lymph node metastasis (N=455)			3.689	0.055
NO	126 (55.0%)	104 (46.0%)		
YES	103 (45.0%)	122 (54.0%)		
TNM (N=503)			5.824	0.016
I-III	232(92.4%)	216(85.7%)		
IV	19(7.6%)	36(14.3%)		

**Table 2:** The relationship between FOXK2 and clinicopathologic characteristics in the validated cohort.

	Case(n)	FOXK2 fold^a^	P-value
Gender			
Male	27	6.0(3.0-8.5)	0.153
Female	71	4.0(2.0-7.0)	
Age			
<55	75	5.5(2.0-8.5)	0.401
≥55	23	5.0(2.5-8.0)	
Tumor Size			
< 2 cm	50	3.5(2.0-5.0)	0.001*
≥2 cm	48	6.8(2.5-10.0)	
LNM			
Yes	62	5.0(2.0-7.5)	0.504
No	36	5.0(2.5-8.0)	
TNM Stage			
I+II	62	4.0(2.0-6.0)	0.013*
III+IV	36	7.0(3.0-9.5)	

*Indicated statistical significance (P<0.05). ^a^Median of relative expression according to HSCORE system, with 25th-75th percentile in parenthesis. LNM, lymph node metastasis.

## Abbreviations

3-MA: 3-Methyladenine
FOXK2: The forkhead box class K 2
FOXO3: Forkhead Box O3
PTC: papillary thyroid carcinoma
ULK1: Unc-51 Like Autophagy Activating Kinase 1
VPS34: Phosphatidylinositol 3-kinase
