# FOXK2 promotes the proliferation of papillary thyroid cancer cell by down-regulating autophagy: Erratum

**DOI:** 10.7150/jca.80215

**Published:** 2022-12-30

**Authors:** Songze Li, Pengliang Wang, Hao Ju, Tiantong Zhu, Jingwen Shi, Ying Huang

**Affiliations:** 1 Department of Ultrasound, Shengjing Hospital of China Medical University, Shenyang, Liaoning 110004, China.; 2 Department of Anesthesiology, Cancer Hospital of China Medical University, Liaoning Cancer Hospital &Institute, Shenyang, Liaoning 110122, China.; 3 Department of Gastroenterology, Tianjin Medical University Cancer Hospital, City Key Laboratory of Tianjin Cancer Center and National Clinical Research Center for Cancer, Tianjin, China.

In the original version of our article, there was an error in Fig. 3K. Specifically, the representative image of shNC BHT-101 cells in Figure 3K is incorrect. The correct image is provided below. This correction will not affect the results and conclusions. The authors apologize for any inconvenience this may have caused.

## Figures and Tables

**Figure 3:**
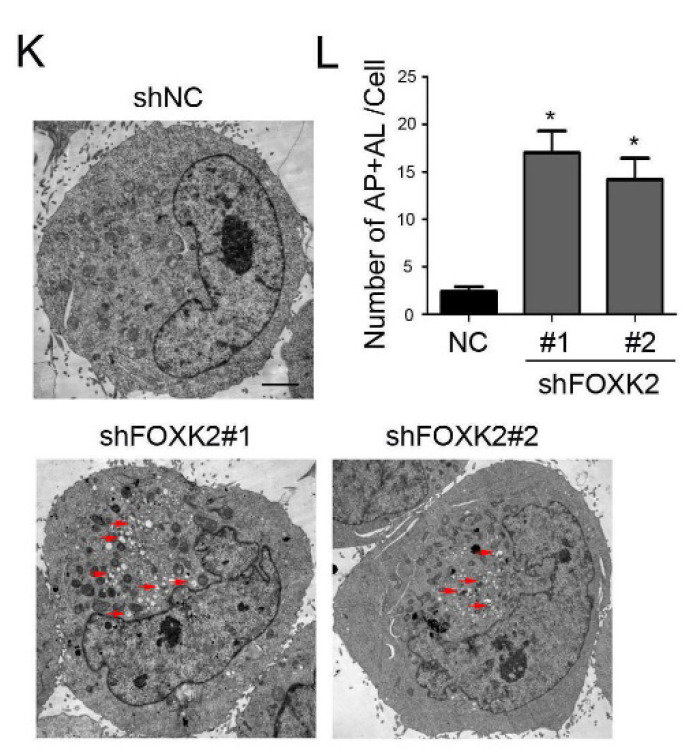
Knockdown of FOXK2 promoted autophagy of PTC cells. (K) Representative electron microscopic image of autophagic vesicles in BHT-101 cells transfected with the indicated shRNA. Scale bars, 500 nm. (L) Electron microscopic quantification of autophagic vesicles in BHT-101 cells transfected with the indicated shRNA. Data are presented as mean±s.e.m. from 3 independent experiments; *P < 0.05.

